# Influence of a Silane Coupling Agent and MWCNTs on the Structural and Durability Performance of CFRP Rebars

**DOI:** 10.3390/ma19010106

**Published:** 2025-12-28

**Authors:** Woo Sung Yum, Do Young Kwon, Yong Sik Chu

**Affiliations:** Climate and Energy Research Division, Research Innovation Headquarters, Korea Institute of Ceramic Engineering & Technology (KICET), 101, Soho-Ro, Jinju 52851, Republic of Korea; wsyum@kicet.re.kr (W.S.Y.); kdy515@kicet.re.kr (D.Y.K.)

**Keywords:** CFRP rebars, epoxy matrix modification, silane coupling agent, MWCNT (multi-walled carbon nanotubes), durability, fire resistance

## Abstract

This study investigates the influence of silane coupling agents and multi-walled carbon nanotubes (MWCNTs) on the mechanical, durability, and thermal performance of CFRP rebars manufactured using a pilot-scale pultrusion process. The incorporation of additives extended epoxy working time without causing adverse viscosity effects during processing. Silane-modified CFRP rebars exhibited the highest mechanical performance, achieving a tensile strength of approximately 2649 MPa, an elastic modulus of 156 GPa, and improved bond strength with concrete, which is attributed to enhanced fiber–matrix interfacial adhesion. MWCNT-modified rebars showed slightly lower tensile strength but demonstrated superior thermal resistance, retaining the highest proportion of mechanical properties after exposure to 250 °C due to matrix reinforcement and crack-bridging effects. No significant degradation was observed under simulated marine exposure, while gradual reductions (up to ~7%) occurred in alkaline environments, with silane-modified rebars showing the greatest durability. These findings provide mechanistic insights and practical guidelines for optimizing epoxy formulations to enhance the structural and long-term performance of CFRP rebars.

## 1. Introduction

With the acceleration of global warming, the frequency of extreme weather events has continued to increase, leading to rapid deterioration in the durability and service life of social infrastructure [1,2]. At the same time, the construction industry has been experiencing continuous growth in the scale and height of structures, further emphasizing the need for high-performance and multifunctional materials that can overcome the limitations of conventional construction materials [3,4,5]. Recent studies have also indicated that increasing structural scale and performance requirements significantly influence design constraints and construction costs, thereby highlighting the necessity of advanced materials capable of ensuring enhanced durability, resilience, and multifunctionality in modern infrastructure systems [6].

Concrete exhibits excellent compressive strength but is inherently weak in tension, whereas steel reinforcement provides high tensile and shear resistance but is vulnerable to corrosion. Even though concrete and steel work well together due to their similar thermal expansion and strong interfacial bonding, steel corrosion is still one of the most serious durability problems in reinforced concrete structures [7,8]. In chloride rich environments, corrosion can progress rapidly, resulting in cracking, section loss, and bond deterioration, which severely compromise structural safety and long-term performance [9]. Moreover, corrosion of embedded steel is difficult to detect and challenging to repair, making the issue particularly critical for large-scale or high-rise structures.

To address these limitations, extensive research has been directed toward the development of carbon fiber-reinforced polymer (CFRP) rebars as a potential alternative to conventional steel reinforcement [10,11]. Among various FRP systems, carbon fiber-reinforced polymer (CFRP) rebars exhibit superior tensile strength, stiffness, and durability compared to glass fiber reinforced polymer (GFRP) and basalt fiber reinforced polymer (BFRP), making them particularly suitable for applications requiring high load-bearing capacity and long-term durability in aggressive environments. CFRP rebars offer several advantages, including high tensile strength, low density, and excellent resistance to corrosion, thereby reducing the self-weight of structures and significantly enhancing durability [12,13]. In addition, their resistance to corrosion, chloride attack, and carbonation provides substantial benefits in terms of long-term serviceability.

Despite these advantages, CFRP rebars have notable limitations, including a lower elastic modulus and higher cost relative to steel. Furthermore, comprehensive verification of their structural applicability, including bond strength with concrete, durability under various environmental exposures (such as marine and alkaline conditions), and fire resistance remains essential [14,15]. Recent studies have shown that the long-term durability of CFRP is strongly governed by matrix degradation and fiber–matrix interfacial stability, particularly under alkaline environments and elevated temperatures [16,17].

Meanwhile, the performance of CFRP rebars is strongly influenced by the epoxy matrix used during manufacture, as epoxy governs fiber integration and plays a critical role in determining mechanical and durability related properties such as tensile strength, elastic modulus, bond strength, environmental resistance, and fire performance [18,19]. Consequently, numerous studies have explored the use of various epoxy types and additives to improve the mechanical properties of CFRP rebars [20]. In particular, silane coupling agents have been widely reported to enhance fiber–matrix interfacial bonding, while multi-walled carbon nanotubes (MWCNTs) have been shown to improve matrix stiffness, thermal stability, and crack resistance in epoxy-based composites. However, because the types of additives are numerous and their effects vary significantly with dosage and processing conditions, further systematic studies remain necessary.

In previous research, CFRP rebars were manufactured using a pilot-scale pultrusion facility, and the fiber count, epoxy ratio, and curing conditions were optimized, resulting in rebars with a tensile strength of approximately 2100 MPa and an elastic modulus of around 150 GPa [21]. However, key structural and durability characteristics such as bond performance, resistance to aggressive environments, and high-temperature degradation behavior have not been sufficiently investigated. Moreover, most existing studies focus on resin-level or laboratory-scale specimens, and systematic investigations on full-scale CFRP rebars incorporating functional additives under realistic manufacturing conditions remain limited.

Therefore, in this study, the previously developed CFRP rebars were evaluated for their fundamental mechanical properties, including tensile strength, elastic modulus, and bond strength. In addition, silane coupling agents and multi-walled carbon nanotubes (MWCNTs) were incorporated into the epoxy matrix to enhance performance, and their effects on the mechanical properties, durability behavior, and fire resistance of the CFRP rebars were examined.

## 2. Materials and Experimental Methods

### 2.1. Materials and Sample Preparation

A variety of materials were used in this study, including carbon fibers, glass fibers, bisphenol-F–based epoxy resins, a silane coupling agent, and multi-walled carbon nanotubes (MWCNTs). The carbon fiber used for the CFRP rebars was H2550K (Hyosung, Seoul, Republic of Korea), while the glass fiber used to form the surface ribs was a 4400 TEX product manufactured by CPIC (Weihai, China). The epoxy matrix consisted of two bisphenol-F–based resins, KFR-5121 and KFH-9581LV (Kukdo Chemical, Seoul, Korea). The silane coupling agent incorporated as an additive was 3-(trimethoxysilyl)propyl methacrylate (Sigma-Aldrich, St. Louis, MO, USA), and the MWCNTs were CNT MR99 supplied by Carbon Nanotech (Pohang, Republic of Korea). Note that carbon fibers were used as the primary reinforcement in the CFRP rebars, governing the tensile strength and elastic modulus of the composite. Glass fibers were locally applied only for surface rib formation to enhance bond performance with concrete and to stabilize the rib geometry during the pultrusion process. In addition, basic properties of raw materials are shown in Table 1.

**Figure 1:**
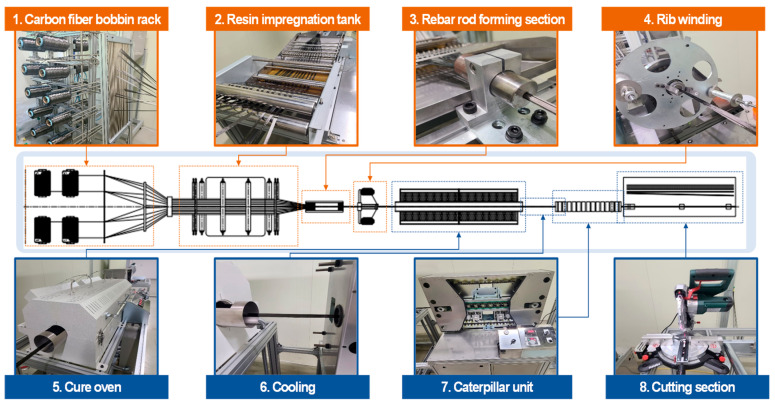
Manufacture Process of CFRP rebars Using a Pilot-Scale Pultrusion Facility.

**Figure 2:**
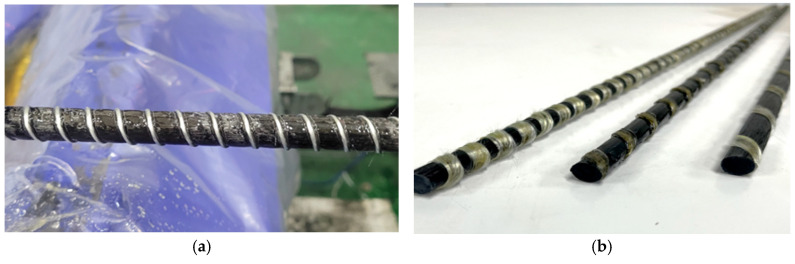
CFRP Rebar manufacture: (**a**) CFRP Rebar during manufacture, and (**b**) Completed CFRP rebar products.

CFRP rebars with a nominal diameter of 10 mm (D10) were manufactured using a pilot-scale pultrusion facility. As shown in Figure 1, the CFRP rebars were fabricated using a pilot-scale pultrusion facility equipped with a dedicated rib-winding section. Carbon fibers impregnated with epoxy resin were first consolidated into a core CFRP rod in the rod forming section. Subsequently, glass fibers were continuously wound around the surface of the CFRP rod to form the rib structure. The rib geometry was stabilized by controlling the winding tension of the glass fibers and synchronizing the winding speed with the pultrusion speed, allowing uniform rib spacing and consistent rib height along the rebar length. Both the core CFRP rod and the rib-forming glass fibers were impregnated with the same epoxy resin system and subsequently cured simultaneously in the curing oven. This co-curing process enabled chemical and mechanical integration at the bar–rib interface, resulting in strong interfacial bonding and preventing rib detachment during handling and subsequent concrete casting. Through this process, CFRP rebars with stable rib geometry and enhanced bond performance were successfully manufactured (See Figure 2).

Chu et al. investigated the effects of epoxy resin content, impregnation temperature, curing temperature, and pultrusion speed to manufacture CFRP rebars achieving a tensile strength of 2100 MPa and an elastic modulus of 150 GPa, and identified the optimal manufacturing conditions. In the present study, these optimized parameters were adopted to fabricate CFRP rebars. The optimal manufacture conditions for the CFRP rebars are summarized in Table 2 [21].

To investigate the influence of additives on the physical properties of the rebars, a silane coupling agent and MWCNTs were incorporated into the epoxy matrix. The mix proportions used for each formulation are shown in Table 3. The addition contents of the silane coupling agent and MWCNTs were determined based on reported effective ranges in previous studies and preliminary experimental evaluations. The selection criteria included resin viscosity, dispersion stability, fiber impregnation behavior, and compatibility with the pultrusion process. Additive contents that caused excessive viscosity increase or dispersion instability were excluded to ensure stable CFRP rebar fabrication [22,23,24,25]. The silane coupling agent and MWCNTs were added at 1% and 0.5% by weight of the epoxy, respectively, while the “Epoxy with Silane & MWCNT” formulation contained both additives simultaneously.

### 2.2. Experimental Methods

To examine the effects of additives on the characteristics of the epoxy matrix, workability and viscosity tests were performed. The viscosity of the epoxy was measured in accordance with ASTM D4440 using a rotational rheometer (TA instruments, New Castle, Delaware, DE, USA) [26]. Epoxy mixtures prepared according to the designated formulations were equilibrated to a target temperature of 45 °C and tested with a concentric-cylinder geometry. Viscosity was continuously recorded to evaluate its variation over time.

The working time of the epoxy resin was assessed following ASTM D2471 [27]. Epoxy mixtures prepared according to Table 2 were placed in a beaker and positioned on a 45 °C hot plate. The samples were monitored continuously from the gelation onset until flowability was lost, and the elapsed time was recorded as the working time.

The tensile strength and elastic modulus of the CFRP rebars were measured in accordance with KS F ISO 10460 [28]. The manufactured rebars were cut into 1600 mm lengths, and both ends were anchored using metal sockets filled with epoxy resin to prevent premature failure during testing. The effective cross-sectional area was measured, and strain gauges were attached along the specified gauge length to monitor deformation. Tensile tests were conducted using a universal testing machine (WOOJIN, Seoul, Republic of Korea); the maximum load recorded in the load–displacement curve was taken as the tensile strength, and the initial slope of the stress–strain curve was used to determine the elastic modulus. Three specimens were tested for each mixture, and the average values were reported.

Bond strength between the CFRP rebar and concrete was evaluated in accordance with ASTM D7913/D7913M [29]. Concrete specimens with dimensions of 200 mm × 200 mm × 200 mm were cast, and a 1300 mm-long CFRP rebar was embedded at the center with an embedment length of 50 mm. After 28 days of curing, the free end of the rebar was gripped in a tensile testing machine, while the concrete block was restrained using a reaction frame. The maximum pullout load was recorded and used to calculate the average bond strength. Three specimens were tested for each mixture.

Fire resistance was evaluated by comparing the tensile strength and elastic modulus before and after high-temperature exposure. The CFRP rebars were placed in a heating chamber and tested once the chamber temperature reached 250 °C. Tensile strength and elastic modulus were determined using the same criteria described above. Three specimens per mixture were tested, and average values were used for comparison.

Durability performance was assessed by immersing the CFRP rebars in simulated chloride and alkaline environments and measuring changes in tensile strength and elastic modulus over time. A 5% NaCl solution, approximating seawater concentration, was used to simulate chloride environment. Two alkaline solutions were used: (1) the ASTM D7705 solution, consisting of 118.5 g Ca(OH)_2_, 0.9 g NaOH, and 4.2 g KOH per liter of distilled water; and (2) a calcium hydroxide solution (3.7 g Ca(OH)_2_ per liter) prepared to more closely replicate concrete pore solution [30]. Both solutions exhibited a pH of approximately 13, confirming their suitability for simulating highly alkaline environments. The use of two different alkaline solutions in this study was motivated by previous findings indicating that the ASTM D7705 solution does not accurately represent the chemical environment inside concrete and often produces excessively severe degradation results [30]. Consequently, many researchers have adopted Ca(OH)_2_ solutions to more realistically simulate the pore solution of concrete. For this reason, both alkaline solutions were included in the present study. The CFRP rebars were immersed in each solution, and tensile strength and elastic modulus were measured after exposure periods of 1, 2, 3, and 6 months to evaluate degradation behavior.

## 3. Results

### 3.1. Changes in Epoxy Properties with Additive Incorporation (Working Time and Viscosity)

**Figure 3:**
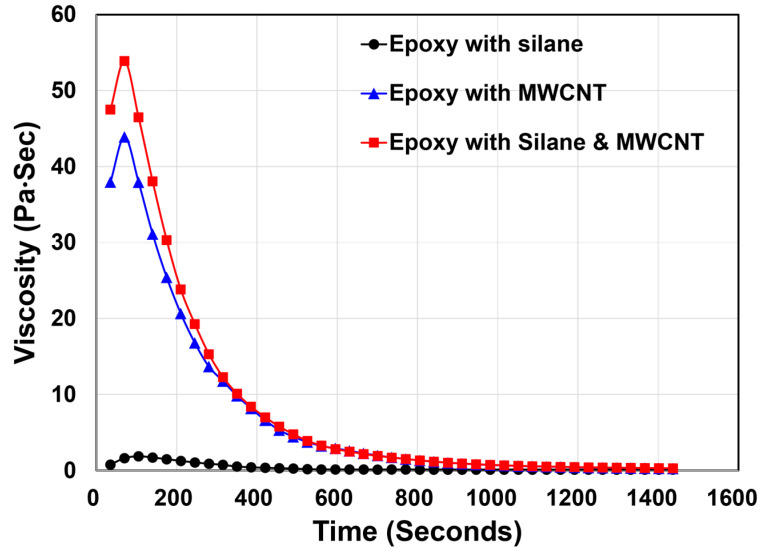
Viscosity Results for Epoxy Mixture proportions.

The working time and viscosity results for epoxy mixtures containing different additives are summarized in Table 4 and Figure 3. All mixtures exhibited working times exceeding 8 h, meeting the minimum requirement for handling and processing. The Epoxy with Silane mixture showed a working time of 12.5 h, while the Epoxy with MWCNT mixture exhibited a longer working time of 16 h, approximately 3.5 h greater than the silane only mixture. Considering that silane and MWCNT were added at 1% and 0.5% by weight of epoxy, respectively, these results indicate that MWCNT plays a more significant role in extending the working time [31,32]. The Epoxy with Silane & MWCNT mixture showed the longest working time due to its higher overall additive content (1.5%). These findings confirm that the incorporation of additives does not reduce the working time of the epoxy and is unlikely to cause workability issues.

The viscosity results likewise reflect the influence of each additive. The Epoxy with Silane mixture maintained a low viscosity of approximately 5 Pa·s or less throughout the test period, demonstrating the most stable flow behavior. In contrast, the Epoxy with MWCNT mixture exhibited a sharp increase in viscosity during the initial stages, reaching a peak of approximately 40 Pa·s at around 120 s [31]. This was followed by a gradual decrease as dispersion progressed. The Silane & MWCNT mixture showed the highest initial viscosity (≈50 Pa·s) and a similar decreasing trend over time. The increased viscosity observed in epoxy with Silane & MWCNT is attributed to enhanced intermolecular interactions and physical network formation, which restrict resin flow. In particular, these observations suggest that MWCNTs are the primary factor responsible for the viscosity increase due to their high aspect ratio and strong particle interaction effects within the epoxy matrix [32].

In prearticular, CFRP rebar manufacture processing using a pilot-scale pultrusion facility, epoxy is mixed for approximately 10 min prior to fiber impregnation. Given the observed reduction in viscosity over time, it is expected that all mixtures would achieve sufficiently low and stable viscosity during mixing, indicating that the additives at the selected dosages will not interfere with the manufacturing process of CFRP rebars.

### 3.2. Mechanical Properties of CFRP Rebars

The tensile strength, elastic modulus, and bond strength of CFRP rebars incorporating different additives are summarized in Table 5. For comparison, CFRP rebars manufactured with unmodified epoxy in a previous study exhibited a tensile strength of 2100 MPa and an elastic modulus of 150 GPa [21].

Among the three mixtures, the CFRP rebars produced with the Epoxy with Silane formulation demonstrated the highest overall mechanical performance, particularly in terms of tensile strength and bond strength. This enhancement is primarily attributed to the improved fiber–matrix interfacial adhesion induced by the silane coupling agent, which promotes chemical bonding at the interfaces between the epoxy matrix and both carbon and glass fibers, thereby facilitating more efficient stress transfer under mechanical loading [22,33].

The Epoxy with MWCNT mixture exhibited a slightly lower tensile strength than the Epoxy with sample, while maintaining comparable elastic modulus and bond strength values. The contribution of MWCNTs is mainly associated with matrix reinforcement and microcrack-bridging effects, which enhance stiffness and damage resistance of the epoxy matrix rather than directly strengthening the fiber–matrix interface.

In contrast, the Epoxy with Silane & MWCNT rebars showed the lowest tensile strength and bond strength among the additive-modified systems. This result suggests that the simultaneous incorporation of silane and MWCNTs resulted in an antagonistic rather than synergistic effect, which may be attributed to competition between the silane molecules and MWCNTs at the fiber–matrix interface, as well as increased resin viscosity that limited effective fiber wetting and uniform impregnation during the pultrusion process [34,35].

Despite these differences, all additive-modified CFRP rebars exhibited higher tensile strength and elastic modulus than the epoxy-only rebars reported in the previous study, indicating the overall effectiveness of epoxy modification strategies. Additionally, all samples exceeded the minimum bond strength requirement of 15 MPa for structural applications, confirming their suitability for practical use.

### 3.3. Evaluation of CFRP Rebar Performance After High-Temperature Exposure

**Figure 4:**
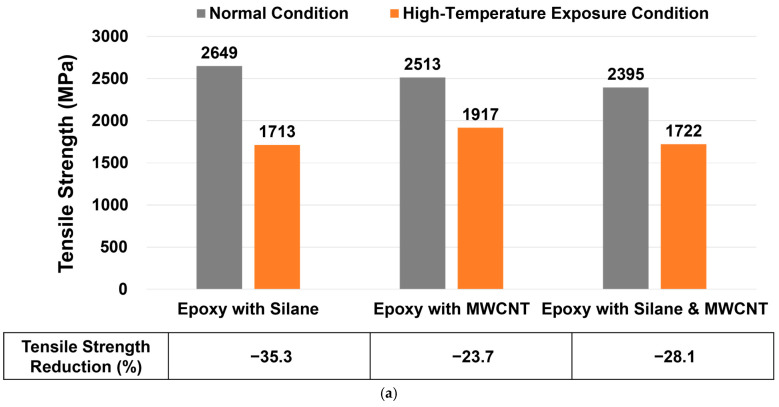

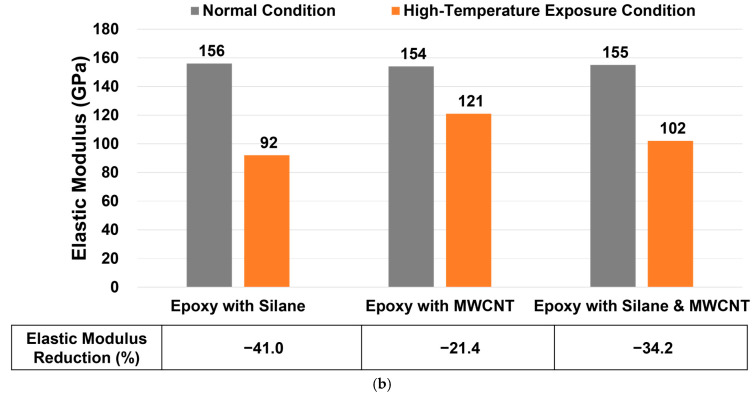
Mechanical Properties of CFRP Rebars Before and After High-Temperature Exposure: (**a**) Tensile Strength and (**b**) Elastic Modulus (mean ± standard deviation).

The tensile strength and elastic modulus of the CFRP rebars before and after high-temperature exposure are presented in Figure 4. Although the reduction ratios varied among the mixtures, a consistent trend was observed based on the type of additive incorporated.

Before exposure, the rebars modified with silane, MWCNT, and the combination of both additives exhibited decreasing tensile strength in that order. After exposure to 250 °C, however, the trend reversed. The reduction in mechanical properties was highest for the Silane modified rebars, followed by the Silane & MWCNT mixture, while the MWCNT only rebars exhibited the smallest reduction. These results indicate that MWCNT provides the greatest enhancement in high-temperature resistance among the additives tested.

The superior thermal stability of the MWCNT-modified rebars can be attributed to the high thermal decomposition temperature and heat dissipation characteristics of carbon nanotubes, which help mitigate thermally induced matrix degradation [36,37]. In contrast, the greater reduction observed in the Silane-modified rebars is likely due to partial thermal degradation of the silane coupling agent, which typically begins degrading within the 200–300 °C range [38].

Although no standardized criteria exist for evaluating strength retention of CFRP rebars after high-temperature exposure, maintaining 60–80% of the original tensile properties is generally considered acceptable. Based on this criterion, all mixtures in the present study retained adequate mechanical performance after exposure, with the MWCNT-modified formulation demonstrating the highest thermal resistance.

### 3.4. Performance Evaluation of CFRP Rebars Under Various Exposure Conditions

**Figure 5:**
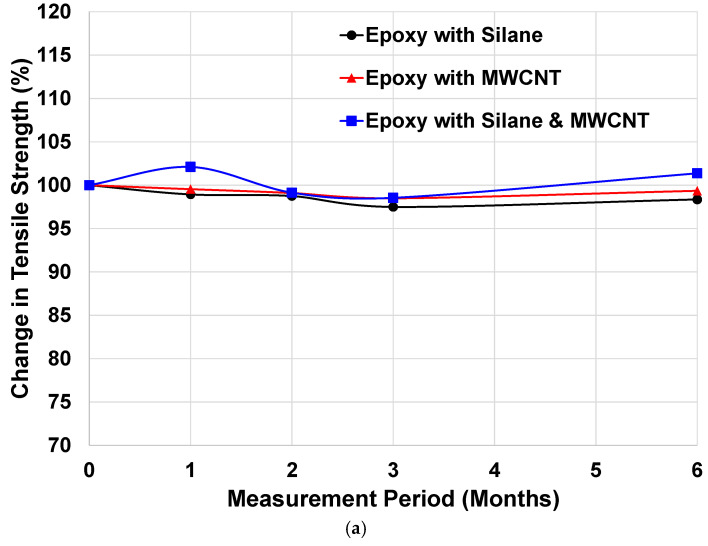

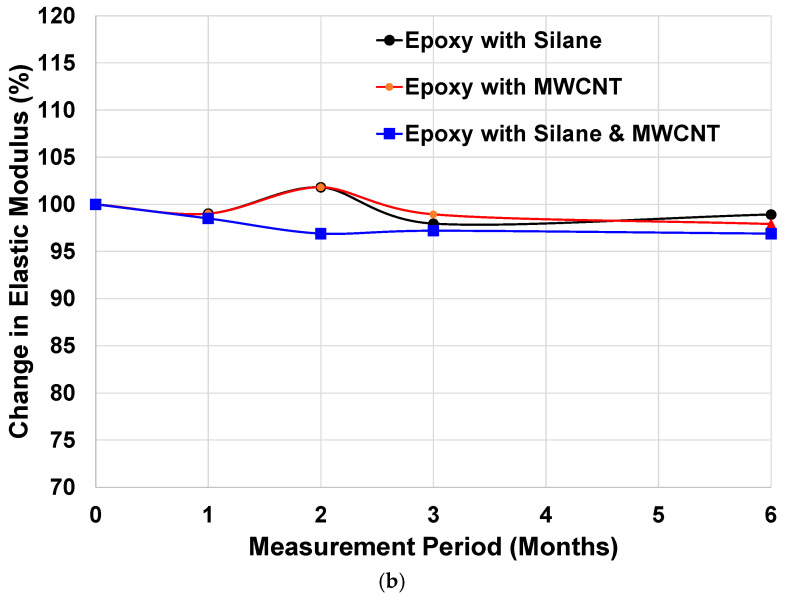
Changes in Mechanical Properties of CFRP Rebars Exposed to a Marine Environment: (**a**) Tensile Strength and (**b**) Elastic Modulus.

**Figure 6:**
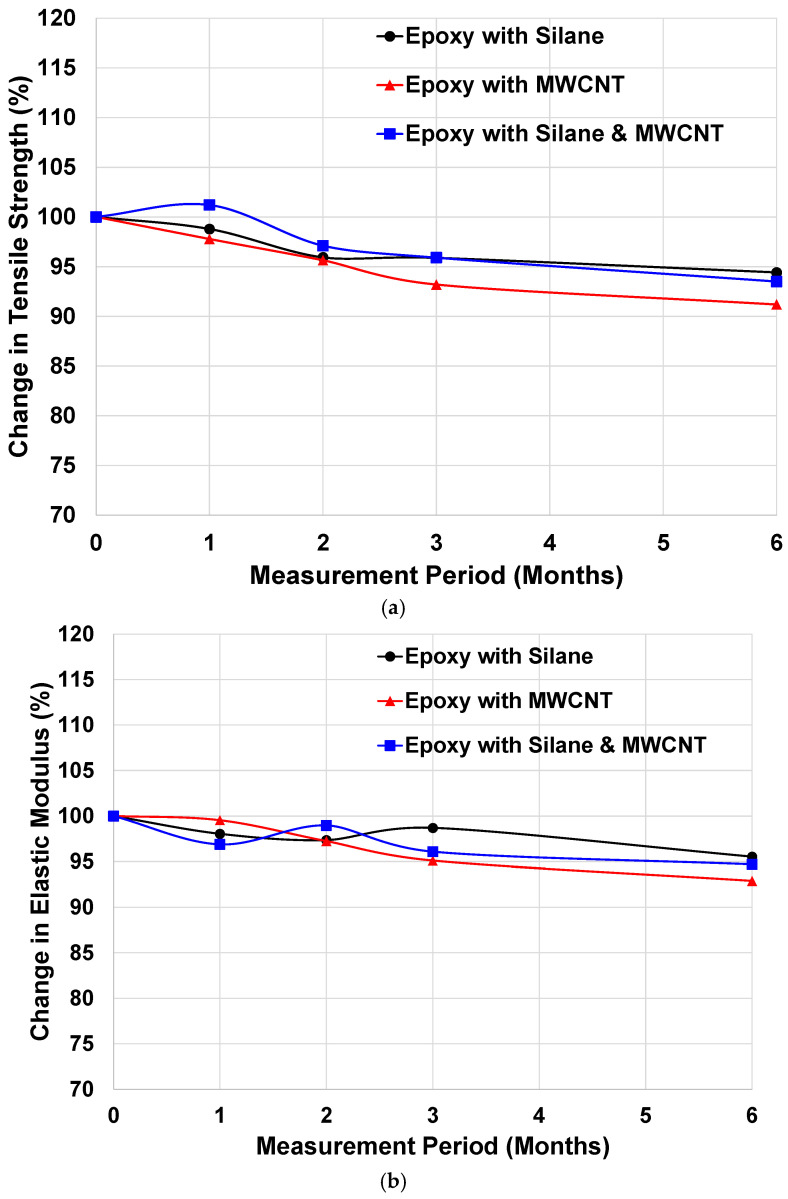

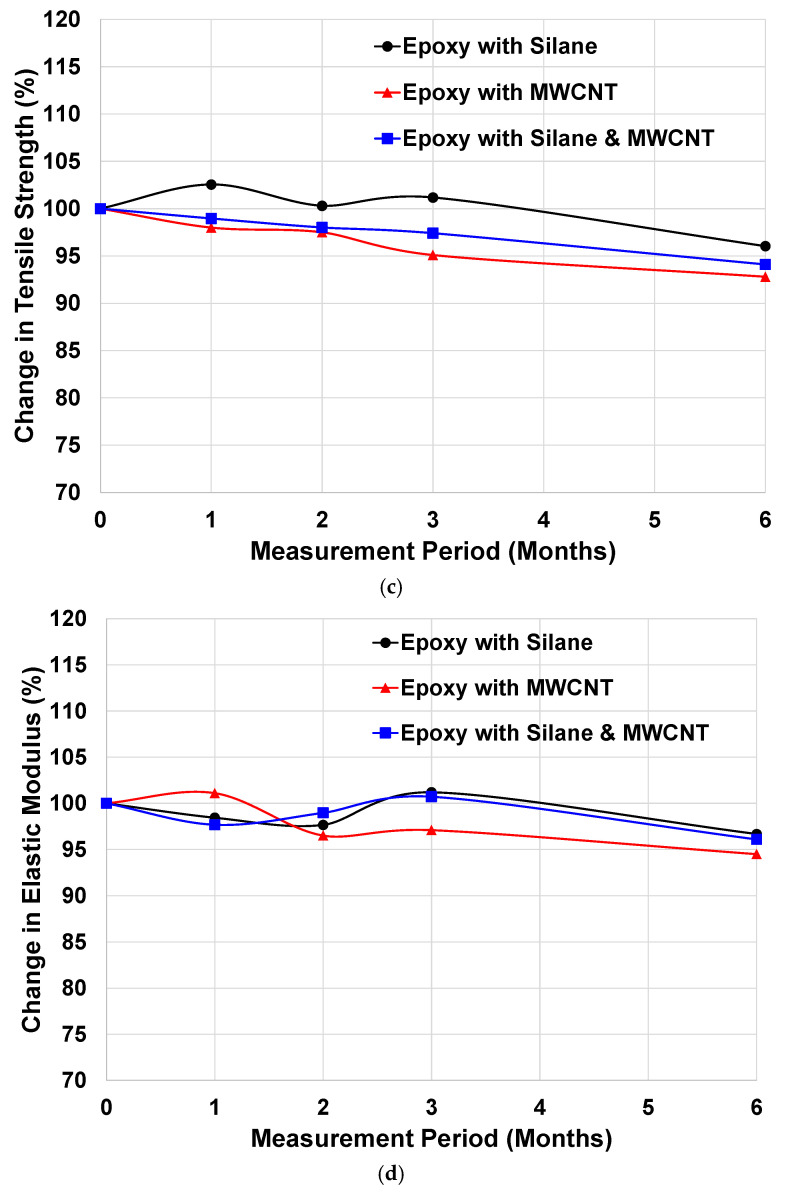
Changes in Mechanical Properties of CFRP Rebars Exposed to Two Different Alkaline Environments: (**a**) Tensile Strength (ASTM Solution), (**b**) Elastic Modulus (ASTM Solution), (**c**) Tensile Strength (Ca(OH)_2_ Solution), and (**d**) Elastic Modulus (Ca(OH)_2_ Solution).

The tensile strength and elastic modulus of the CFRP rebars under different exposure conditions are presented in Figure 5 and Figure 6.

For specimens exposed to the simulated marine environment (See Figure 5), no significant changes in mechanical properties were observed for any mixture or exposure duration. All variations remained within ±2% of their initial values, a range that is commonly considered within experimental scatter rather than indicative of true material degradation. This behavior reflects the inherent corrosion resistance of CFRP materials, as carbon fibers are immune to chloride-induced corrosion and the epoxy matrix shows minimal chemical interaction with chloride ions. Therefore, CFRP rebars are expected to maintain their durability in marine environments [39,40].

Subsequently, two different alkaline environments were examined, and the results are summarized in Figure 6. Unlike the marine exposure results, all samples exhibited gradual performance reductions over time, with maximum decreases of approximately 7% [39]. Regardless of alkaline solution type, the degradation trends were consistent among the additive groups. The largest reduction occurred in the Epoxy with MWCNT rebars, whereas the Epoxy with Silane rebars showed the smallest decrease.

These observations are consistent with the mechanical properties measured prior to exposure (see Table 4). Although the MWCNT-modified rebars initially exhibited higher tensile performance than the unmodified epoxy, they experienced the greatest degradation under alkaline attack. This response is attributed to the fact that MWCNTs primarily act as physically dispersed fillers and do not form strong chemical bonds with the epoxy matrix. In contrast, silane coupling agents chemically react with both the epoxy matrix and the fiber surfaces, enhancing interfacial adhesion and improving resistance to chemical attack. As a result, silane-modified rebars d This response is attributed to the fact that MWCNTs primarily act as physically dispersed fillers and do not form strong chemical bonds with the epoxy matrix demonstrated superior durability in alkaline environments.

The severity of degradation also differed between the two alkaline solutions. CFRP rebars immersed in the ASTM D7705 solution exhibited more pronounced performance loss than those exposed to the Ca(OH)_2_ solution [30]. This outcome aligns with previous studies reporting that although both solutions exhibit similar pH values (≈13), the ASTM D7705 solution contains significantly higher concentrations of Na^+^ and K^+^ ions, which accelerate chemical degradation and do not accurately represent the pore solution of concrete [30,41,42,43].

Despite the observed reductions, the overall mechanical losses remained relatively small. Therefore, major durability concerns in actual concrete structures are not expected under comparable exposure conditions; however, long-term exposure studies are still recommended to fully validate in-service performance.

From a long-term performance perspective, the incorporation of silane coupling agents and MWCNTs influences the durability of CFRP composites through different mechanisms. Silane coupling agents primarily improve long-term interfacial stability by strengthening chemical bonding at the fiber–matrix interface, thereby reducing interfacial debonding and moisture-assisted degradation during prolonged environmental exposure.

In contrast, MWCNTs contribute to long-term performance mainly by reinforcing the epoxy matrix, bridging microcracks, and inhibiting crack propagation, which delays damage accumulation under sustained mechanical and environmental loading. Similar mechanisms have been reported in previous studies, where nanofillers were shown to enhance long-term mechanical stability and durability of polymer composites [44].

When combined, the effectiveness of these two fillers depends on their dispersion state and interfacial interactions. Excessive viscosity increases or competitive interactions at the fiber–matrix interface may limit their synergistic potential, highlighting the importance of optimized filler content for achieving durable composite performance.

## 4. Conclusions

In this study, CFRP rebars incorporating various additives were fabricated, and their performance was systematically evaluated. Mechanical properties, including tensile strength, elastic modulus, and bond strength with concrete, were initially examined. Subsequently, the effects of high-temperature exposure and different environmental conditions (marine and alkaline) were assessed. The major findings are summarized as follows:

1. The incorporation of silane coupling agents and MWCNTs increased the working time of the epoxy matrix, and although MWCNTs initially increased viscosity, all mixtures reached stable viscosity levels suitable for pultrusion processing.
2. All additive-modified CFRP rebars exhibited higher tensile strength and elastic modulus than those fabricated with unmodified epoxy. Silane provided the greatest mechanical enhancement by improving fiber–matrix interfacial adhesion, while the combined use of silane and MWCNTs resulted in an antagonistic effect due to interfacial competition and increased viscosity.
3. After exposure to 250 °C, CFRP rebars showed reductions in mechanical properties; however, MWCNT-modified rebars retained the highest proportion of their original strength and stiffness, demonstrating superior thermal resistance through matrix reinforcement and crack-bridging effects.
4. No meaningful degradation was observed in simulated marine environments, confirming the inherent durability of CFRP rebars under chloride exposure. In contrast, gradual performance reductions occurred under alkaline conditions, with silane-modified rebars showing the greatest resistance due to enhanced interfacial stability.
5. Rebars exposed to the ASTM D7705 alkaline solution experienced more severe deterioration than those immersed in Ca(OH)_2_, supporting previous findings that the ASTM solution is significantly more aggressive and not fully representative of real concrete pore environments.

## Figures and Tables

**Table 1:** Basic Properties of Raw Materials.

Raw Material	Tensile Strength (MPa)	Elastic Modulus (GPa)	Density (g/cm^3^)	Remark
Carbon Fiber	4500–5000	230–240	~1.80	Primary reinforcement
Glass Fiber	2000–3000	70–75	~2.55	Rib formation
Epoxy resin	60–80 *	3.0~3.5 *	~1.20	Matrix material

* Tensile strength and elastic modulus of cured epoxy resin.

**Table 2:** Manufacture Conditions for CFRP Rebars.

Resin to Harder Ratio	Impregnation Zone Temperature (°C)	Curing Oven Temp (°C)						Production Speed (cm/min)	Curing Time (min)
47:53	45	120	180	120	130	170	180	3	30

**Table 3:** Mixture Proportions of Epoxy with Additive Incorporation (wt%).

Sample Label	Epoxy Resin		Additive		Sum
	Resin	Harder	Silane Coupling Agent	MWCNT	
Epoxy with Silane	47.14	51.86	1.0	0.0	100.0
Epoxy with MWCNT	47.60	51.90	0.0	0.5	
Epoxy with Silane & MWCNT	46.90	51.60	1.0	0.5	

**Table 4:** Working Time Results for Epoxy Mixture proportions.

Sample Label	Working Time (Hours)
Epoxy with Silane	12.5
Epoxy with MWCNT	16
Epoxy with Silane & MWCNT	20

Note: The working time refers to the period during which the epoxy resin remains processable during the impregnation and pultrusion process.

**Table 5:** Mechanical Properties of CFRP Rebars Manufactured with Different Epoxy Mixture Proportions (mean ± standard deviation).

Sample Label	Tensile Strength (MPa)	Elastic Modulus (GPa)	Bond Strength (MPa)
Epoxy with Silane	2649 (49)	156 (8)	17.0 (1.0)
Epoxy with MWCNT	2513 (25)	154 (9)	16.5 (0.7)
Epoxy with Silane & MWCNT	2395 (72)	155 (8)	15.5 (0.5)

## Data Availability

The original contributions presented in this study are included in the article. Further inquiries can be directed to the corresponding author.
